# Increased ^18^ F-FDG uptake in denervated muscles in a case of Parsonage-Turner syndrome

**DOI:** 10.1186/s12883-023-03328-x

**Published:** 2023-07-26

**Authors:** Ping-I Chiang, Chiung-Mei Chen

**Affiliations:** 1 Center for Medical Education in English, Poznan University of Medical Sciences, Poznan, 60-512 Poland; 2 Linkou Medical Center, Department of Neurology, Chang Gung Memorial Hospital, Taoyuan, Taiwan; 3 Department of Neurology, College of Medicine, Chang Gung University, Taoyuan County, 33305 Taiwan

**Keywords:** Parsonage-Turner syndrome, Neuralgic amyotrophy, 18F-FDG uptake, Denervated muscle

## Abstract

**Background:**

Parsonage-Turner Syndrome (PTS) is a rare brachial plexopathy characterized by the sudden onset of pain in the shoulder girdle followed by upper limb weakness. PTS is frequently under-recognized or misdiagnosed as other more common neurological disorders presenting in a similar fashion, such as cervical radiculopathy which may require surgical intervention. Accurate diagnosis and prompt management implicate a good prognosis. Although electrophysiological studies are considered the most important for evaluating peripheral nerve injuries, it usually takes time, up to 3 weeks after the initial insult of the nerve for electromyogram (EMG) and nerve conduction studies (NCS) to display abnormalities. In the cases of PTS, especially when initial EMG/NCS and magnetic resonance neurography (MRN) results are inconclusive, ^18^ F-FDG positron emission tomography and computed tomography (^18^ F-FDG PET-CT) may be useful in helping the early detection of muscle denervation.

**Case presentation:**

A 60-year-old right-handed Taiwanese woman presented with sudden onset of intense and sharp left shoulder girdle pain without radiating to the arm, followed by muscle weakness of her left arm in abduction and elevation 3 days after the onset of pain. A detailed neurological examination and EMG and NCS suggested the clinical diagnosis of left brachial plexopathy. MRN imaging revealed no significant abnormality. ^18^ F-FDG PET-CT showed increased uptake in denervated muscles (supraspinatus, deltoid, and biceps muscles). Treatment with oral prednisolone and physiotherapy significantly improved pain and muscle weakness.

**Conclusions:**

We present increased ^18^ F-FDG uptake in denervated muscles detected by ^18^ F-FDG PET-CT. ^18^ F-FDG PET-CT may serve as an adjunct examination to evaluate PTS, which has been suggested previously but rarely reported.

## Background

Parsonage –Turner syndrome (PTS), also known as Neuralgic Amyotrophy (NA), is an uncommon peripheral neuropathy characterized by an acute onset of severe pain, which is usually unilateral and involves the neck or upper extremities, followed by muscle weakness with slow but gradual recovery [1]. The causes of PTS have not been clearly established; however, certain precipitating factors such as infection, vaccination, trauma, and autoimmune diseases have been reported. Recent vaccination has been estimated to account for 4.3% of cases [2].

The clinical presentation together with thorough examinations are usually sufficient to establish the diagnosis of PTS; however, in atypical cases or when the physician is not familiar with the condition, misdiagnosis can occur, which may lead to unnecessary diagnostic and therapeutic interventions or delayed management. Although most cases of PTS recover gradually, patients may be left permanently impaired if not recognized and treated properly. The use of corticosteroids in the acute phase of PTS can provide significant pain relief and fasten the recovery process [2, 3]. Therefore, accurate diagnosis and prompt treatment is important for PTS to have a good prognosis. Previous reports have suggested that increased 18-fluorodeoxyglucose (^18^ F-FDG) uptake in denervated muscles detected by ^18^ F-FDG positron emission tomography and computed tomography (^18^ F-FDG PET-CT) may help in diagnosis of PTS [4, 5].

## Case presentation

A 60-year-old right-handed Taiwanese woman presented with sudden onset of intense and sharp left shoulder girdle pain without radiating to the arm 4 days prior to visiting our clinic on 17th, Nov, 2022, followed by muscle weakness of her left arm in abduction and elevation 3 days after the onset of pain. Tracking back her history, she received her first dose of COVID-19 vaccine, Moderna mRNA-1273, on 11th, June, and second dose on 20th, July, 2022, without significant side effects other than mild localized pain around the injection site in left deltoid muscle. She had no history of neurologic diseases or allergies and denied recent trauma or infection. The shoulder pain was not significantly relieved by conservative measures, such as massage, and non-steroid anti-inflammatory agents. The severity of weakness progressed rapidly over the next three days, and she could only raise her arm up to 45 degrees. A detailed neurological examination showed decreased muscle power in the supraspinatus (Medical Research Council grade, 3), deltoid (2), biceps (4), brachioradialis (4), and supinator (4), on the left side. The contralateral arm and bilateral lower extremities demonstrated full range of motion, full strength, and normal sensation without any pain. Deep tendon reflexes were normal and there were no sensory deficits.

**Fig. 1:**
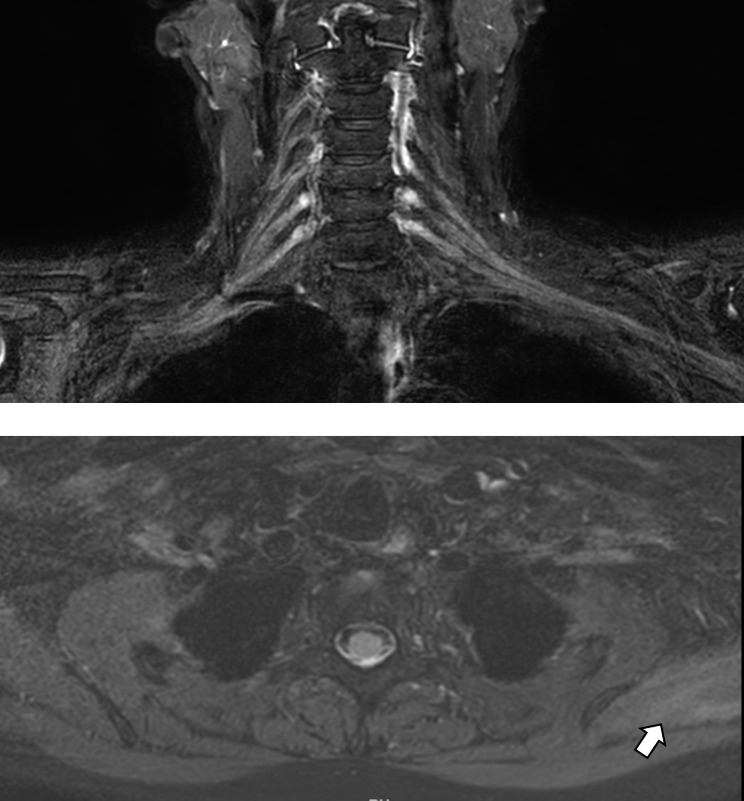
The images of magnetic resonance neurography (upper panel) and muscle magnetic resonance image (lower panel). The brachial plexus shows no significant abnormality. The white arrow indicates slightly increased T2 signal in the left supraspinatus muscle

**Fig. 2:**
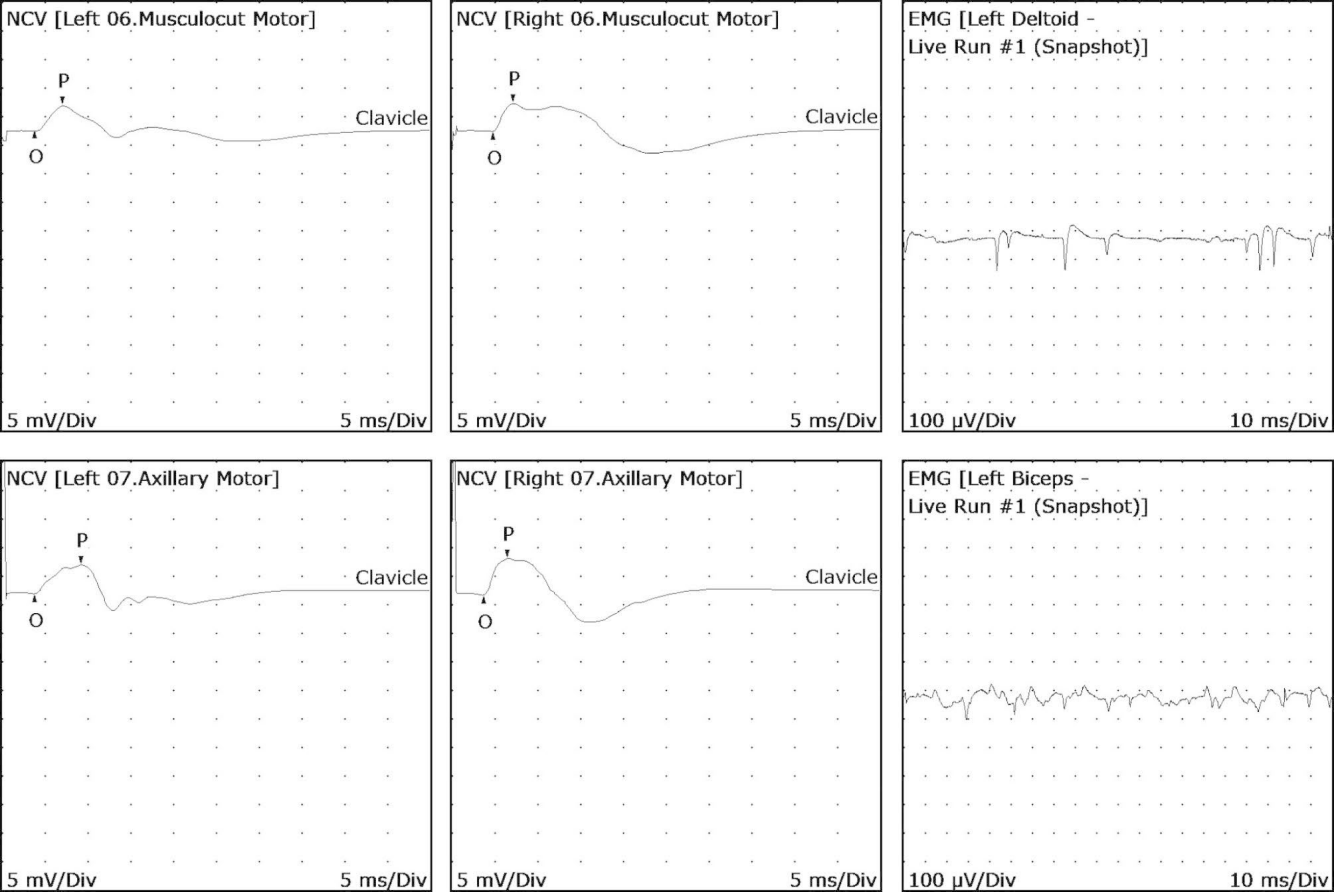
The electromyogram (EMG) and nerve conduction velocity studies (NCS) of the patient one month after the onset of symptoms. NCS only shows slightly decreased amplitudes of left musculocutaneous and left axillary compound motor action potentials. EMG of shows positive waves in left deltoid and left biceps

**Fig. 3:**
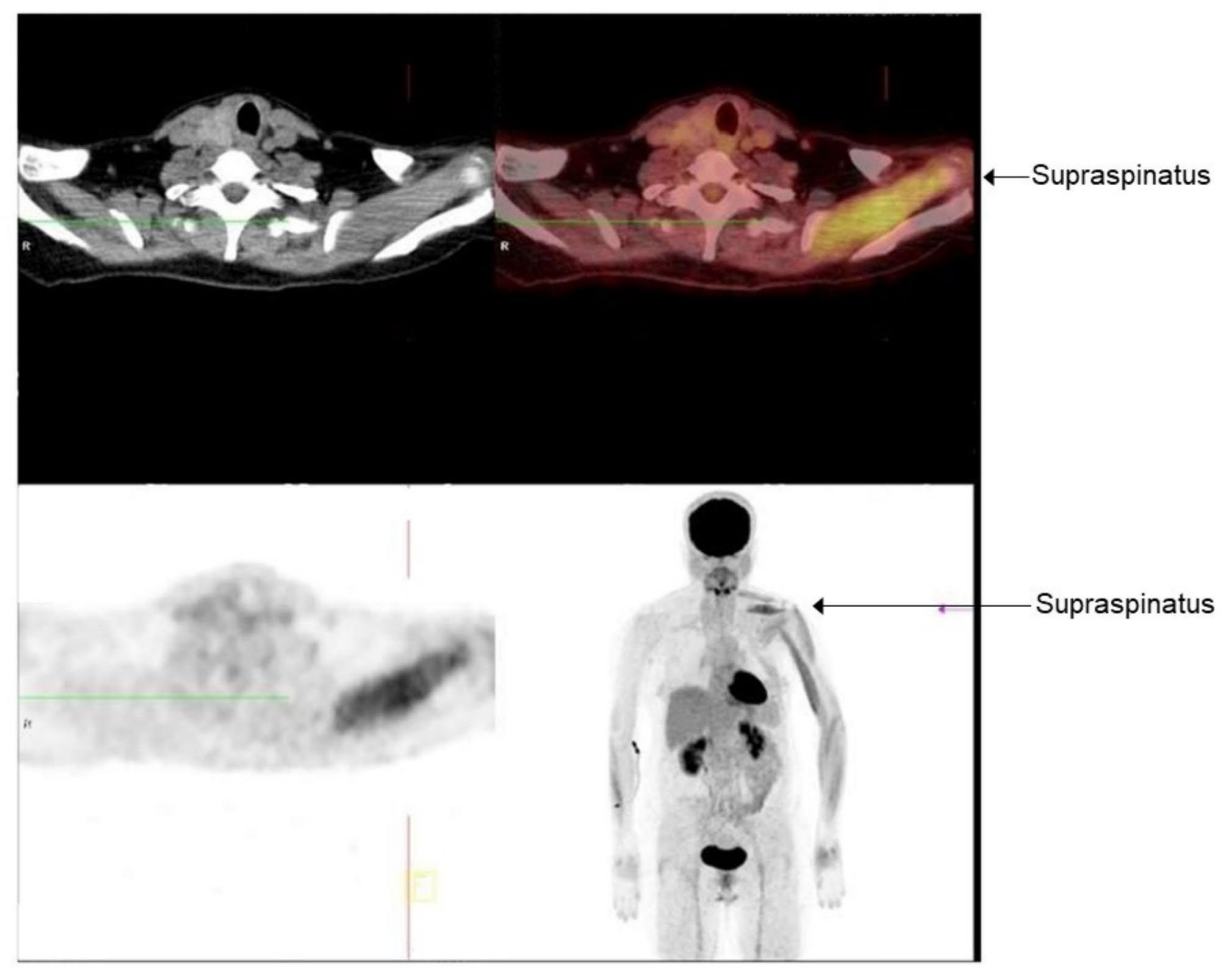
The positron emission tomography–computed tomography (PET-CT) scan of the patient. The image displays diffused areas of increased radioactivity uptake (score 1) in the supraspinatus, deltoid and bicep muscles of the left upper limb

Blood workup showed normal complete blood count, tumor markers (AFP, CA125, CA19-9, CA15-3, CEA, SCC, CYFRA21-1), free T4, and TSH levels. Magnetic resonance imaging (MRI) of the cervical spine without contrast was performed on day 6, which showed degenerative spondylolisthesis grade 1 in cervical vertebra (C) 5 and C6, mild protrusion of intervertebral disc between C4 and C5, and tiny marginal osteophytes with no significant bone lesions or displaced fractures. Magnetic resonance neurography (MRN) imaging revealed no significant abnormality in left brachial plexus but slightly increased T2-weighted signal in left supraspinatus was noted (Fig. 1). Electromyogram (EMG) and nerve conduction studies (NCS) were conducted at two weeks and one month, respectively, after the onset of initial symptoms. The first EMG/NCS showed normal sensory nerve action potentials and borderline low compound motor action potentials of the left musculocutaneous and axillary nerves (lower compared to the right), along with decreased recruitment of the deltoid muscle and increased polyphasic waves of long duration in the rhomboid, deltoid, biceps, supraspinatus and supinator muscles on the left side. The results of the second EMG/NCS were similar to the initial one with the additional findings of active denervation in the deltoid and bicep muscles, indicated by fibrillations and positive waves on EMG (Fig. 2). The EMG/NCS results showed left brachial plexopathy mainly involving left upper trunk. The ^18^ F-FDG PET-CT scan obtained one month later showed diffused areas of increased radioactivity uptake (score 1) in the supraspinatus, deltoid and bicep muscles of the left upper limb (Fig. 3).

Subsequent treatment with oral prednisolone (40 mg/day) was initiated on day 5 and continued for 12 days without tapering. The level of muscle weakness remained stationary for the first 10 days following treatment and gradually started to improve afterwards. Recovery of muscle strength by 50% was subjectively observed by the patient in the first month and up to 90% of recovery was noted 3 months after the onset of first symptom. Six months later, muscle power had almost resumed to its prior state.

## Discussion

The clinical and EMG/NCS findings of our patient suggests the diagnosis of PTS. However, other more common conditions that may cause acute shoulder pain and upper limb muscle weakness must be ruled out. Differential diagnoses include cervical radiculopathy, shoulder joint pathology, mononeuritis multiplex, multifocal motor neuropathy, and peripheral nerve entrapment. Other causes of brachial plexopathy such as trauma, post-radiation, primary or metastatic tumor, infection, vasculitis, and hereditary neuropathy with liability to pressure palsy should be ruled out. Blood examinations, imaging tests (High-resolution ultrasonography (HRUG), MRI, ^18^ F-FDG PET-CT) may assist in excluding conditions such as cervical disc pathology, and infectious or neoplastic etiologies. Initially, cervical radiculopathy was suspected in our case; however, MRI revealed minor disc protrusion between the cervical vertebral bodies of 4 and 5 (C4-5) and tiny marginal osteophytes that were more consistent with mild degenerative changes rather than C5 radiculopathy caused by acute cervical disc compression. Recent advanced studies in nerve imaging have shown focal abnormalities in proximal nerve segments of brachial plexus in 75–80% of PTS patients [1]. It is noted that MRN images of a proportion of PTS patients are normal, similar to the MRN of our patient. As a result of the clinical, imaging and EMG/NCS findings, we concluded that PTS seemed to be the most likely diagnosis [1, 2].

To rule out the possibility of malignant tumors and metastatic infiltration of the brachial plexus, ^18^ F-FDG PET-CT was performed on our patient. Although the MRN showed only suspected increased T2-weighted signal in left supraspinatus, it is interesting to note that on ^18^ F-FDG PET-CT imaging, affected muscles that experienced weakness (supraspinatus, deltoid and biceps muscles) showed a diffuse pattern of increased signal intensity. Increased radioactive uptake in tissues compared to baseline reflects an increase in glucose metabolism, which may indicate the presence of neoplasms, however, active inflammation and tissue repair could also be the cause. In a preliminary study, ^18^ F-FDG uptake was increased in denervated muscles 8-days post-nerve resection in rat models, which confirms glucose hypermetabolism in denervated muscles [6]. In another study conducted in rat models, glucose hypermetabolism began 2 days after nerve injury and lasted up to 12 weeks, with maximal increase at week 1 after denervation. ^18^ F-FDG signal intensity from the PET scans were strongly correlated with the severity of nerve injury. The mechanism for the increase in glucose metabolism after nerve injury is not fully understood; however, one of the postulated mechanisms is spontaneous muscle contraction after nerve injury. Molecular pathways that are thought to be involved, include the mammalian target of rapamycin (mTOR) and apoptosis pathway. In that study, the authors found increase in phosphorylated-mTOR and mTOR expression which peaked along with maximum 18 F-FDG uptake. Significant increased B-cell lymphoma 2 (Bcl-2)-associated X protein (Bax)/Bcl-2 expression (ratio of apoptotic activity) was observed 7 days after denervation [7]. This suggests that the intensity of the increased 18 F-FDG uptake in the affected muscles of our patient may reflect the extent of nerve injury. Similar to our case, increased 18 F-FDG uptake in affected muscles in cases of brachial neuritis have also been reported [4, 5]. Although electrophysiological studies are considered the most important for the evaluation of peripheral nerve injuries, it usually takes time, up to 3 weeks after the initial insult of the nerve for EMG/NCS to display abnormalities. Similarly, the EMG of our case also only showed mild denervated changes in affected muscles 14 days after the onset of symptoms. As the literature has shown that PET imaging displays increased uptake in denervated muscles as early as 2–8 days after denervation, we proposed that PET imaging may detect muscle denervation earlier than EMG that typically does not show significant changes within 2 weeks following the onset of neuralgic amyotrophy. In the case of PTS, especially when initial EMG/NCV and MRN results are inconclusive, ^18^ F-FDG PET-CT may be useful in helping the early detection of muscle denervation, as in the case of PTS.

Although we did not perform HRUG in our patient, it is worth noting that HRUG can be a valuable tool in assessing PTS. Nerve swelling is the most common sonographic finding in PTS, other pathologic HRUS findings that are also observed in PTS include nerve constriction (incomplete or complete) and fascicular entwinement [8]. HRUG is readily available, lacks contraindications, and has shown good diagnostic utility when performed by experienced examiners. However, ultrasound imaging of the plexus itself can be challenging for non-specialized examiners due to limited accessibility beneath the clavicle. In such cases, MRN and 18 F-FDG PET-CT may be more sensitive in detecting muscle denervation.

The clinical features of our case are in concordance with most cases of PTS in literature [1]. The initial intense pain usually occurs prior to the onset of muscle weakness and does not correlate with the localization of the paresis [9]. In our case, there were no sensory symptoms associated with muscle weakness, which is reported in up to 78.4% of patients with suspected PTS [2]. In PTS, the most commonly affected nerves are the long thoracic and/or suprascapular nerve; however, any part of the brachial plexus can be involved [2]. Although most cases of PTS post-COVID-19 vaccination present with the classical form, atypical phenotypes of pure sensory or painless motor-predominant forms [10] and lumbosacral involvement [11] have also been observed. In our case, the time from the antecedent vaccination to the onset of PTS is 115 days, which is substantially longer than those of most cases [10–24]; however, a recent case series reported cases that occurred 3 months after vaccination [20]. Although it is not feasible to establish a robust causal relationship between PTS and COVID-19 vaccination, but given that there were no other precipitating factors that could better explain for the clinical presentation of our previously healthy patient, the mRNA Moderna vaccine seems to be the most probable trigger. This observation suggests that the onset of PTS after a predisposing event could vary greatly from person to person, which may reflect the complex interplay between genetic predisposition and environmental factors as the etiologies of PTS.

In a large case series of 246 patients with PTS, most patients recovered from severe to mild residual muscle weakness in the course of months to a few years, with or without corticosteroid treatment. However, when comparing the corticosteroid-treatment patients with the untreated group, the duration of muscle weakness was significantly shorter [2]. In 2009, a Cochrane review suggested that administration of oral prednisolone during the first month of an attack could shorten the duration of pain and also accelerate recovery [3]. Similarly, administration of oral prednisolone to our patient when PTS was suspected led to substantially improved muscle weakness one month later. It is important to note that most cases of PTS post-COVID vaccination also reported the use of corticosteroids, which led to improvement in muscle weakness and near full recovery within the course of 1 to 6 months [10–13, 15, 17–24]. Our case report demonstrates that identification and accurate diagnosis of PTS is crucial because while debilitating, the prognosis of PTS is usually favorable if treated and managed promptly with treatments including corticosteroid medications and physiotherapy.

## Conclusion

We present a case of PTS which occurred post-COVID-19 vaccination and improved after corticosteroid administration and physiotherapy. Early recognition of PTS minimizes unnecessary diagnostic and therapeutic interventions, and allows for earlier symptomatic relief as it is usually steroid-responsive. Our case suggests that ^18^ F-FDG PET-CT may be useful as an adjunct functional imaging assessment for the diagnosis and evaluation of peripheral nerve injury in PTS.

## Data Availability

All data included in this study are available upon reasonable request by contact with the corresponding author.

## Abbreviations

PTS: Parsonage –Turner syndrome
^18^F-FDG: 18-fluorodeoxyglucose
^18^F-FDG PET-CT: ^18^ F-FDG positron emission tomography and computed tomography
MRI: Magnetic resonance imaging
MRN: Magnetic resonance neurography
EMG: Electromyogram
NCS: Nerve conduction studies
mTOR: mammalian target of rapamycin
Bcl-2: B-cell lymphoma 2
Bax: Bcl-2-associated X protein
C: cervical vertebra
HRUG: High-resolution ultrasonography
